# Reconstruction du ligament croisé antérieur selon la technique de Mac Intosh modifiée par Jaeger: étude rétrospective de 80 cas au service d’orthopédie et traumatologie CHU Habib Bourguiba, Sfax, Tunisie

**DOI:** 10.11604/pamj.2022.43.27.34763

**Published:** 2022-09-19

**Authors:** Mourad Aoui, Fedi Dahech, Nizar Sahnoun, Rekik Mohamed Ali, Ahmed Mahjoub Jarboui, Mariem Ghribi, Yosr Hentati, Hassib Keskes

**Affiliations:** 1 Service de Chirurgie Orthopédique et Traumatologique, Centre Hospitalier Universitaire Habib Bourguiba, Sfax, Tunisie,; 2 Centre Sectoriel de Médecine et de Sciences de Sport, Sfax, Tunisie,; 3 Service de Radiodiagnostic et d´Imagerie Médicale, Centre Hospitalier Universitaire Hedi Chaker, Sfax, Tunisie

**Keywords:** Ligament croisé antérieur, reconstruction, bandelette ilio-tibiale, Mac Intosh, Anterior cruciate ligament, reconstruction, ilio-tibial band, Mac Intosh

## Abstract

Les études récentes de la chirurgie du ligament croisé antérieur (LCA) tendent à mieux contrôler la stabilité rotatoire mais la reconstruction du LCA reste toujours un problème d'actualité. Le but de notre travail est d´évaluer nos résultats sur le plan clinique et radiologique de notre série ou nous avons adopté la technique de reconstruction anatomique de LCA au Fascia lata selon Mac Intosh modifiée par J.H Jaeger. Cette étude intéresse 80 patients issus d´une série continue entre 2005 et 2019. Tous les patients ont été évalués selon le score IKDC selon l´échelle Lysholm et Tegner. Tous nos patients sont classés excellents avec une moyenne de 92. Les douleurs résiduelles latérales occasionnelles ont été rapportées par 8 patients. Le Jerk résiduel dans notre série était de 2.5%. L´analyse radiologique n´a pas montré de ballonnisation ni de phénomènes arthrosiques au dernier recul. La reconstruction du LCA est une intervention largement pratiquée qui s´est fiabilisée au cours des temps. Mais la notion de ressaut rotatoire (Jerk test) exige une ténodèse latérale « retour externe » surtout chez les sportifs.

## Abstract (en)

Recent studies about anterior cruciate ligament (ACL) surgery have focused on a better control of rotational stability, but ACL reconstruction is always a topical issue. The purpose of our study was to evaluate clinical and radiological outcomes after the use of the technique for anatomical reconstruction of the ACL with fascia lata, according to Macintosh technique modified by J.H Jaeger. We conducted a study of a continuous series of 80 patients between 2005 and 2019. All patients were assessed according to the International Knee Documentation Committee (IKDC) score and the Lysholm and Tegner scale. All our patients were rated as excellent with an average score of 92. Occasional lateral residual pain was reported by 8 patients. Residual jerk accounted for 2.5%. Radiological examination did not show ballooning or arthrosis at the last follow-up. Anterior cruciate ligament reconstruction is a common procedure that has become more reliable over time. But pivot shift (Jerk test) requires a lateral tenodesis “external rotation”, especially in athletes.

## Introduction

La rupture du ligament croisé antérieur (LCA) est une des lésions les plus fréquentes en traumatologie du genou chez les sportifs. Cette lésion est très invalidante, vu l´instabilité qu´elle entraîne, pouvant empêcher la reprise de l´activité sportive et même évoluer vers une gonarthrose secondaire [1]. La réparation chirurgicale occupe largement le devant de la scène thérapeutique chez le sportif [2]. Le but de notre étude était d´évaluer nos résultats clinico-radiologiques de la reconstruction du ligament croisé antérieur utilisant une bandelette ilio-tibiale avec un retour externe systématique.

## Méthodes

**Cadre de l´étude:** il s´agit d´une étude rétrospective réalisée entre 2005 et 2019 portant sur 80 patients opérés au service d´orthopédie et traumatologie du CHU Habib Bourguiba de Sfax pour une laxité antérieure chronique du genou.

**Collecte des données:** la collecte des patients s'est faite à partir des registres médicaux. Des fiches d'exploitation préétablies ont été remplies regroupant les paramètres épidémiologiques, cliniques, thérapeutiques et évolutifs, ainsi qu'à la convocation des patients pour évaluer les résultats à long terme. La saisie des données a été effectuée sur une fiche informatisée avec le programme SPSS 13. Certains histogrammes ont été réalisés à l´aide du programme SPSS, d´autres à l´aide du logiciel Microsoft Excel 2017.

**Figure 1:**
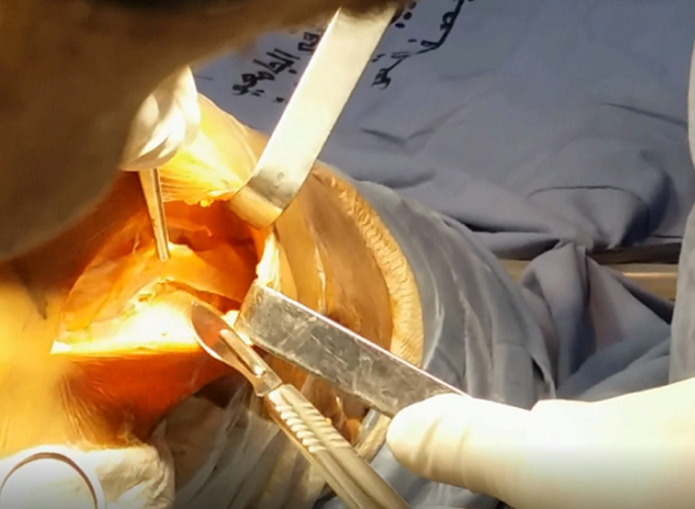
prélèvement du transplant

**Sources de données/mesures:** nous avons recensé l´âge, le sexe, les antécédents des patient, les circonstances de survenue, le mécanisme ainsi les signes fonctionnels et les données de l´examen physique. Tous les patients ont été explorés par une radiographie du genou face et profil et par une imagerie par résonance magnétique (IRM). La technique de choix réalisé pour tous les patients était la technique de Mac Intosh au fascia lata modifiée par Jaeger. Ces modifications ont porté sur le trajet transversal du transplant à la hauteur du condyle fémoral latéral et sur la réalisation d´une plastie de translation antérieure du septum intermusculaire latéral permise par une section longitudinale de celui-ci, le long de son insertion fémorale. Le transplant prélevé avait une largeur de 4 cm à sa partie proximale, 1.5 cm à sa partie distale avec une longueur de 15 cm au minimum, tout en restant attaché au tubercule de Gerdy (Figure 1).

**Figure 2:**
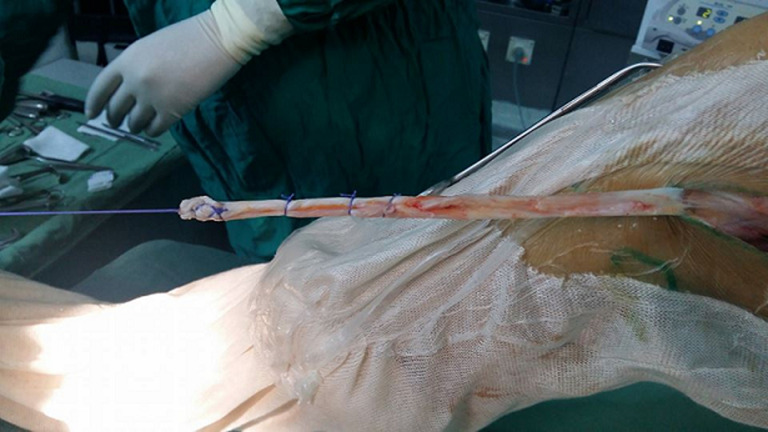
tubulisation du transplant

La partie proximale a été tubulisée par des points inversés afin de faciliter le passage dans les tunnels et pour augmenter le volume du transplant (Figure 2). Le vaste latéral a été décollé au doigt du septum intermusculaire latéral qui sera incisé longitudinalement permettant ainsi la translation de la cloison intermusculaire qui servira par la suite à la fermeture. Le temps arthroscopie a commencé par une exploration du cartilage et des ménisques. Un geste chirurgical approprié a été réalisé en présence d´une lésion méniscale et/ou cartilagineuse. Nous avons veillé à ce que le nettoyage de l´échancrure soit de façon de préserver un environnement vasculo-nerveux probablement bénéfique transplant. Le forage du tunnel tibial était progressif, utilisant des mèches de diamètre croissant de 3,2 mm, 4,5 mm et 6mm. Son trajet aboutissait dans le pied du LCA rompu. Avant de procéder à la fixation du transplant, l´isométrie a été vérifiée en extension et en flexion complète de genou. L´absence d´avalement nous a confirmé le bon positionnement de la plastie. La tension du transplant a été réglée par traction manuelle exercée par l´assistant. La fixation du transplant était réalisée soit par une agrafe métallique crantée soit par une vis d´interférence, et parfois par les deux.

**Figure 3:**
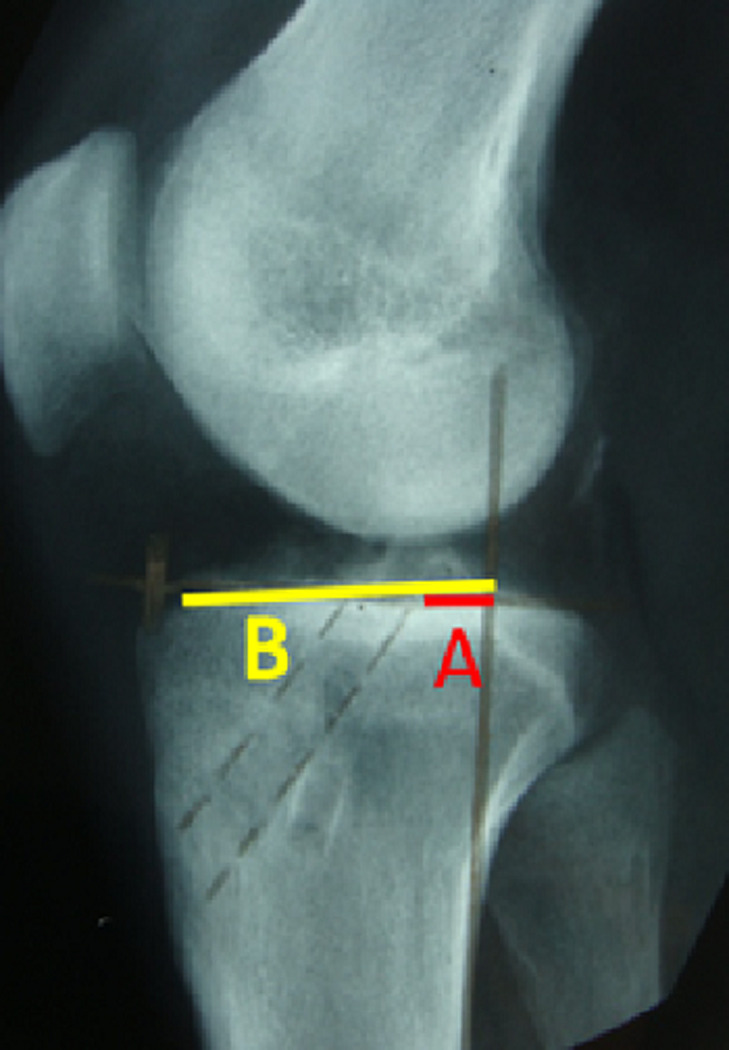
mesure de positionnement du tunnel tibial de profil selon Courage

**Figure 4:**
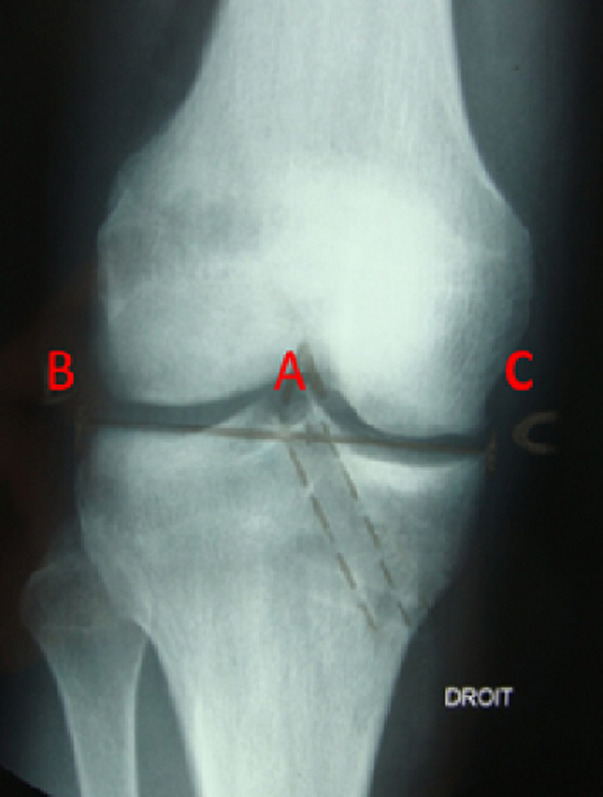
mesure de positionnement du tunnel tibial de face selon Romano

**Figure 5:**
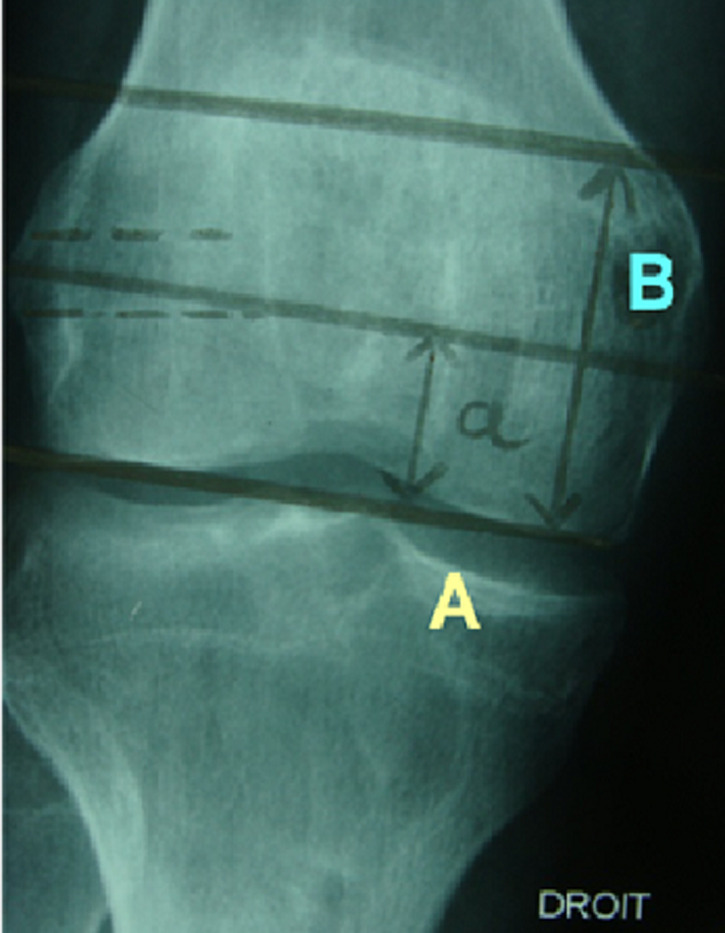
mesure de positionnement du tunnel fémoral de profil selon Zaccherotti et Aglietti

**Figure 6:**
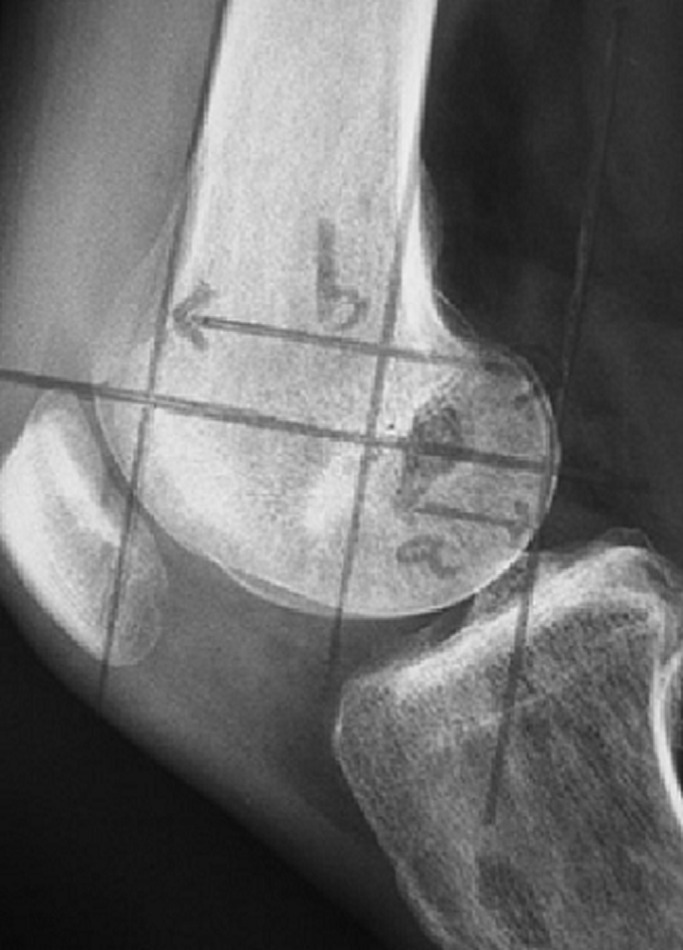
mesure de positionnement du tunnel fémoral de face selon Locker et Vielpeau

Les patients étaient évalués par le score d´over all IKDC (Tableau 1) et Lysholm Tegner [3] (Tableau 2). L´analyse clinique postopératoire a été basée sur des critères subjectifs et objectifs universellement admis [4]. L´évaluation radiologique a étudié essentiellement la survenue d´une arthrose et le positionnement des tunnels. Pour le tunnel tibial, nous avons mesuré les indices de Courage (Figure 3) et de Romano (Figure 4). Au niveau du fémur, le positionnement de tunnel a été déterminé par les indices de Zaccherotti et Aglietti sur la face (Figure 5) et par l´indice de Vielpeau et d´Aglietti sur le profil (Figure 6) [5,6]. Nous avons cherché notamment des signes de ballonnisation ou de dégradation arthrosique.

**Tableau 1:** score overall IKDC

Overall IKDC score	A	B	C	D
Impression subjective: comment fonctionne votre Genou. Échelles de 0 à 3 Influence genou/activité	0	1	2	3
**Signes fonctionnels**				
Douleur	I	II	III	IV
Epanchement	I	II	III	IV
Insécurité	I	II	III	IV
Déboîtement	I	II	III	IV
Mobilité articulaire	A	B	C	D
Flessum	<3°	3-5°	6-10°	>10°
Déficit flexion	0/5°	6-15°	16-25°	>25°
**Laxite (mm)**				
Test de Lachman	- 1 à 2	3 à 5	6 à 10	>10
Arrêt dur mou	Dur		Mou	
Tirroiranterieur	0 à 2	3 à 5	6 à 10	>10
Pivot shift	=	+	+	+
Examen des compartiments	A	B	C	D
Craquements	0	Moyenne	Modéré	Sévère
**Site de prélèvement**				
Douleur, dysesthésie	0	Moyenne	Modéré	Sévère
Radiologie (arthrose mm)				
Pincement interligne	0	>4	2-4	<2
**Test fonctionnel**				
Saut /1 pied (% opposé)	>90	76-90	50-75	<50
**Total**				

**Tableau 2:** le score de Lysholm et Tegner

Instabilité	Douleur	blocage	Gonflement	escaliers	Accroupissements	Boiterie	Cannes
Jamais 25	Jamais 25	Jamais 15	Jamais 10	Jamais 10	Jamais 5	Jamais 5	Jamais 5
Rare 20	Modère 20	Accrochage 10	Activité intense 6	Activité intense 6	Leger handicap 4	Modéré 3	Permanent 2
Fréquent 15	Important 15	Blocage occasionnel 6	Activité courante 2	Activité courante 2	Pas plus 90° 2	Sévère 0	Station debout impossible 0
Occasionnel 10	Marche < 2 km 10	Blocage fréquent 2	Constant 0	Constante 0	Impossible 0		
Souvent 5	Marche > 2 km 5	Blocage aigu à l'examen 5					
Chaque pas 0	Constante 0						
0-64= mauvais 65-84 moyen 85-100 bon/excellent							

## Résultats

L´âge moyen des patients était de 28 ans avec des extrêmes entre 16 et 48 ans. Le sexe-ratio était de 19. Avant la survenue du traumatisme, 30 patients (37%) étaient des sportifs de competition (C), 16 patients (34%) étaient des sportifs de loisir (L) et 16 patients (20%) étaient actifs (A). Seulement 7 patients (9%) étaient sédentaires. Les sports les plus pratiqués étaient de type pivot avec contact (PC): 73 patients étaient des footballeurs (91%), deux patients étaient des handballeurs (3%), trois patients étaient de kick-boxeurs (4%), un patient était judoka (1%) et un patient était basketballeur (1%). Le traumatisme du genou était secondaire à un accident sportif (AS) dans 95% des cas. La douleur et l´instabilité étaient les signes fonctionnels les plus rapportés par les patients. Elles étaient retrouvées dans 87,5% des cas pour la douleur et 55% pour l´instabilité. Les épanchements articulaires étaient rapportés dans la moitié des cas. Trente pour cent des patients présentaient un blocage articulaire. Dans notre série, nous avons noté deux cas de flessum (de 10° et de 30°) et trois cas de déficit de flexion (parmi ces deux, un patient a gardé une mobilité de 0 à 90° malgré 2 mois de rééducation).

L´amyotrophie du quadriceps comparativement au côté controlatéral a été trouvée chez vingt-neuf patients (36%), l´amyotrophie était inférieure ou égale à 2cm, chez trois patients (4%), elle était supérieure à 2cm. Le Lachman était en arrêt dur retardé (+) chez 7 patients (9%) et en arrêt mou (+) chez 70 patients (88%). Trois patients (4%) avaient une laxité très importante: Lachman (+). Le ressaut rotatoire (Jerk test) était présent chez tous les patients; trois patients (4%) avaient un ressaut en ébauche, 60 patients (75%) avaient un ressaut franc et 17 patients (21% des cas) avaient un ressaut explosif. Le tiroir antérieur direct (TAD) était positif dans tous les cas. Neuf patients avaient un TAD à (+), neuf patients avaient un TAD à (+) et 66 patients avaient un TAD à (+). Le tiroir en rotation externe était positif chez 44 patients (55% des cas). Un syndrome méniscal clinique a été retrouvé chez 21% des patients. Les radiographies standards préopératoires ne montraient pas de lésions mis-à-part des signes d´épanchement intra-articulaires chez 41 patients. L´IRM a montré une rupture du LCA dans 97% des cas.

Le délai moyen de prise en charge était de 9 mois avec des extrêmes allant d´un mois à 38 mois. Le recul moyen était de 44 mois avec des extrêmes allant de 6 mois à 14 ans. Trente de nos patients avaient un recul supérieur à 5 ans. Tous nos patients ont eu le même protocole de rééducation physique, avec une durée moyenne de 4,6 mois et des extrêmes allant de 3 à 7 mois. Soixante-huit pour cent de nos patients étaient très contents des résultats de l´opération chirurgicale contre seulement 26% qui étaient simplement contents et six pour cent étaient mécontents. Cinquante-cinq pour cent de nos patients sont satisfaits de leurs cicatrices et 45% sont indifférents. Au dernier recul, 10% seulement de nos patients (8 cas) ont présenté des douleurs modérées et occasionnelles. Nous avons constaté une récupération totale de la mobilité chez tous les patients. Nous avons noté une amyotrophie significative du quadriceps chez deux patients qui étaient non adhérents vis-à-vis de la rééducation. Ces 2 mêmes patients avaient un pivot-shift résiduel. Seulement trois patients ont présenté des hématomes au niveau du site de prélèvement. Concernant la reprise du sport, 73% de nos patients ont repris leurs activités sportives avec le même niveau pré-blessure, 18% des cas ont repris le sport avec un niveau inférieur, 5% ont changé de discipline et 5% ont décidé d´arrêter le sport définitivement.

Tous nos patients ont été classés « excellents » à l´échelle Lysholm et Tegner avec une moyenne d´échelle de Lysholm de 92,45 et des extrêmes allant de 67 à 100. Par ailleurs, la moyenne totale dans la classification Tegner était à: 6,88 avec des extrêmes allant de 3 à 10. Pour le score Overall IKDC, 69% des patients étaient classés IKDC A, 19% sont classés B, 3% IKDC C et aucun cas IKDC D. Pour l´évaluation radiologique l´indice de Romano moyenne était de 0.47±0.09 avec des extrêmes entre 0.31 et 0.66. Le positionnement du tunnel fémoral a été évalué par l´indice de Courage, la moyenne était de 0.49±0.08 avec des extrêmes entre 0.38 et 0.7. L´indice de Courage moyenne était de 0.38 ± 0.06 avec des extrêmes entre 0.3 et 0.5. L´indice de Vielpeau et Cocker, moyenne était de 0.41±0.1 avec des extrêmes entre 0.32 et 0.66. L´indice de Zaccherotti et Aglietti moyenne était de 0.71±0.05 avec des extrêmes entre 0.6 et 0.8. Aucune ballonnisation des tunnels n´a été observée ni signes d´arthrose. Nous n´avons pas détecté de déviation axiale secondaire cliniquement ni sur les radiographies.

## Discussion

Cette étude présente l'intérêt d'être une série homogène de 80 patients porteurs d'une lésion du LCA, tous opérés avec la même technique, par le même chirurgien. La série est comparable à celles publiées dans la littérature en ce qui concerne l´âge, le sexe, le sport, la durée d´instabilité préopératoire, les lésions associées [1,7,8]. Dans notre étude, le recul moyen était de 44 mois. Nous nous sommes fixés un minimum de 6 mois pour l´évaluation des résultats. Nous n´avons pas déploré de cas de raideur articulaire dans notre série mais nous avons remarqué une récupération rapide de l´extension par rapport à la flexion. Ceci s´explique par la souplesse de la bandelette ilio tibiale (BIT) [9,10]. La notion de douleur antérieure retrouvée fréquemment dans les plasties au tendon rotulien (entre 19% et 38% selon les séries) [11], était absente dans notre technique.

Au dernier recul, l´évaluation des laxités a montré un Lachman résiduel inférieur à 5 mm chez tous les patients, sans retentissement sur l´activité sportive. Le ressaut rotatoire a été observé chez deux patients opérés respectivement à 32 et à 40 mois post-traumatique et qui n´ont pas bien adhéré au programme de rééducation et de réadaptation fonctionnelle. Ceci confirme l´effet anti-ressaut rotatoire du retour externe (2.5% de Jerk résiduel dans notre série qui est nettement inférieur à d´autres techniques) [10-13]. Nous avons obtenu 96% de genoux normaux ou presque normaux avec la cotation I.K.D.C et 95% de bons et excellents résultats avec la cotation Arpège. Ces résultats étaient comparables avec ceux retrouvés dans les études [4,14-16]. L´examen clinique n´a pas montré une déviation axiale secondaire au prélèvement du fascia lata, grâce à l´artifice de Jaeger permettant la fermeture du fascia lata, constituant un bon hauban externe du genou agissant à la fois sur la laxité frontale et rotatoire [2]. Le forage du tunnel fémoral de dedans en dehors ou de dehors en dedans reste encore un sujet à discussion. Les auteurs utilisant les ligamentoplasties de type Kenneth Jones (KJ) ou droit interne demi-tendineux (DIDT) prônent le plus souvent la technique de dedans en dehors, en forant un tunnel fémoral borgne du fait du gain de durée opératoire et de la réalisation d´une seule incision.

Chambat [2,17], comme d´autres auteurs [8,18,19], défend la technique de dehors en dedans pour réaliser un tunnel fémoral tel que son bord antérieur se situe au niveau du point isométrique. On sait aussi qu´il n´existe pas de fibres strictement isométriques, et que le transplant sera positionné dans tous les cas dans une configuration de « non isométrie favorable ou défavorable » [18,19]. Selon les caractéristiques anatomiques et biomécaniques du « système 4 barres » constitué par le pivot central, Chambat [2,17] en conclut que seule une « non isométrie favorable » doit être recherchée, c'est-à-dire une position qui met le transplant en tension lors de l´extension; ainsi, comme pour le LCA natif, il existera lors de l´extension un recrutement progressif des fibres, allant des plus antérieures (les plus isométriques) vers les plus postérieure. Pour cela, il faut positionner le transplant au niveau fémoral juste en arrière du point isométrique, car il s´agit de la position anatomique [2,20,21].

Pour réaliser un tunnel idéal, la technique de forage de dehors en dedans nous paraît donc beaucoup plus fiable. De plus, notre technique impose un abord latéral pour le prélèvement du fascia lata, ce qui constitue un argument supplémentaire dans notre choix de forage du tunnel fémoral selon une technique de dehors en dedans [22,23]. L´iatrogénie des ligamentoplasties du LCA est directement causée ou majorée par le prélèvement d´un transplant aux dépends de l´appareil extenseur ou des ischio-jambiers. Une des particularités de notre technique est de respecter complètement le rôle de ces tendons de la stabilisation du genou, ce qui permet d´éviter cette morbidité [24]. Nous déconseillons le débridement arthroscopique intempestif du genou. La préservation des reliquats du ligament rompu est fondamentale dans le phénomène de ligamentisation et la récupération proprioceptive précoce du genou. Ces reliquats contiennent des mécanorécepteurs qui serviront à la reprise plus précoce des capacités proprioceptives [25,26] et participeraient à la néo vascularisation du transplant [27].

En ce qui concerne le fascia lata, des complications relatives au prélèvement du transplant peuvent venir grever les suites opératoires, dans le cas où le défect du fascia lata n´est pas refermé: risque accru de décoaptation externe avec laxité en varus, hernies du vaste latéral [2,28]. La fermeture sans tension du défect engendré par le prélèvement du transplant a toujours été possible du fait de la modification rapportée par J.H. Jeager consistant en une section de la cloison intermusculaire et une translation de la partie postérieure du fascia lata [18,28]. Les patients que nous avons revus ne se plaignaient pas particulièrement du préjudice esthétique des cicatrices. Pour les tunnels osseux, la réduction du diamètre évite les ballonnisations ultérieures, source d´échec et de laxité résiduelle venant compliquer les techniques utilisant le tendon rotulien et les ischio jambiers.

## Conclusion

La ligamentoplastie du LCA par une bandelette ilio-tibiale décrite par Mac Intosh modifié par Jaeger et ses élèves concernant l´artifice de fermeture du hauban externe empêche toute déviation axiale. L´utilisation de l´arthroscopie pour une meilleure exploration intra-articulaire et un bon positionnement des tunnels. Cette plastie mixte où le retour externe se fait de façon systématique exige un respect strict des règles de protection du pivot central et de sa plastie garant d´une récupération fonctionnelle dans un bref délai.

### Etat des connaissances sur le sujet

- La rupture du LCA pose un problème pronostique pour le sportif;
- La ligamentoplastie est une intervention courante;
- La multitude de publications qui comparent les différentes techniques.

### Contribution de notre étude à la connaissance

- L´utilisation de l´arthroscopie pour une meilleure exploration intra articulaire et un bon positionnement des tunnels;
- Cette plastie mixte où le retour externe se fait de façon systématique est à l´ origine de l´efficacité de cette technique;
- Cette technique permet une récupération fonctionnelle dans un délai de 6 mois.
